# Hybrid G/BN@2H-MoS_2_ Nanomaterial Composites: Structural, Electronic and Molecular Adsorption Properties

**DOI:** 10.3390/nano12244351

**Published:** 2022-12-07

**Authors:** Amal Al-Khaldi, Mohamed M. Fadlallah, Fawziah Alhajri, Ahmed A. Maarouf

**Affiliations:** 1 Department of Physics, Institute for Research and Medical Consultations, Imam Abdulrahman Bin Faisal University, Dammam 31441, Saudi Arabia; 2 Department of Physics, Faculty of Science, Benha University, Benha 13518, Egypt; 3 Department of Physics, Faculty of Basic Sciences, The German University in Cairo, New Cairo 13411, Egypt

**Keywords:** transition metal dichalcogenides, boron nitride, graphene, hybrid structures, molecular adsorption, density functional theory

## Abstract

Hybrid structures often possess superior properties to those of their component materials. This arises from changes in the structural or physical properties of the new materials. Here, we investigate the structural, electronic, and gas-adsorption properties of hybrid structures made from graphene/hexagonal boron nitride and 2H-molybdenum disulfide (G/BN@MoS_2_) monolayers. We consider hybrid systems in which the G/BN patch is at the Mo plane (model I) and the S plane (model II). We find that the implanted hexagon of G or BN in MoS_2_ alters its electronic properties: G@MoS_2_ (I,II) are metallic, while BN@MoS_2_ (I) is an n-type conducting and BN@MoS_2_ (II) is semiconducting. We study the molecular adsorption of some diatomic gases (H_2_, OH, N_2_, NO, CO), triatomic gases (CO_2_, NO_2_, H_2_S, SO_2_), and polyatomic gases (COOH, CH_4_, and NH_3_) on our hybrid structures while considering multiple initial adsorption sites. Our results suggest that the hybrid systems may be suitable materials for some applications: G@MOS_2_ (I) for oxygen reduction reactions, BN@MoS_2_ (I,II) for NH_3_-based hydrogen production, and G@MoS_2_ (I) and BN@MoS_2_ (I,II) for filtration of No, Co, SO_2_, H_2_S, and NO_2_.

## 1. Introduction

The dimensionality of material structures plays an important role in determining their physical and chemical properties [1]. Two-dimensional (2D) materials have attracted considerable attention in improving the performance of electronic and optoelectronic devices [2,3]. In recent years, many studies focused on 2D materials such as graphene (G) [4], hexagonal boron nitride (h-BN (BN)) [5], graphitic carbon nitride [6,7], phosphorene [8], MoSi_2_N_4_ [9,10], PdPSe [11], and other materials.

Transition metal dichalcogenides (TMDs, e.g., MoS_2_) are 2D layered structures with many applications in electronics [1]. Due to an intrinsic band gap ranging from 0.4–3.1 eV [12,13], they have recently received much attention. Furthermore, TMDs monolayers can possess high mobility for charge carriers at room temperature, which makes them attractive materials for optoelectronics and energy-harvesting applications [14].

Molybdenum disulfide monolayers, TMD-(MoS_2_), have two phases 2H-MoS_2_ (two layers per hexagonal unit cell) or 1T-MoS_2_ (one layer per trigonal unit cell). Several experimental techniques can change the physical and chemical properties of the MoS_2_ structure: doping [15], surface functionalization by metal atoms [16], ion bombardment [17], and defect formation [18]. Density functional theory (DFT) is widely used to investigate the effect of substitutional doping with nonmetal, halogen, and transition atoms [19,20] on the electronic and magnetic properties of 2H-MoS_2_. The ferromagnetic behavior of Co- [21] and Fe- [22] doped MoS_2_ monolayer were demonstrated. Furthermore, it is found that the substitutional doping of MoS_2_ can enhance its electrochemical catalytic response [23].

Heterostructures have also been studied experimentally and theoretically, as they may help tailor material properties to specific applications. A hybrid made from G patched by BN has been successfully fabricated and was found to possess an electronic gap [24,25,26]. Such periodic structural defects may lead to two enhancements in a material’s electronic properties: (1) they may cause a change in the conducting state of the structure (e.g., semiconductor to metal), because of the redistribution of some of the electronic states resulting from interaction between the constituents of the hybrid, and (2) the interface between the constituents may form electrostatic traps which can be utilized for molecular adsorption properties of the material.

By using a hydrothermal method, heterostructures of MoS_2_/G [27], MoS_2_/WS_2_ [28], MoSSe/MoS_2_ [29], MoS_2_/WSe_2_ [30], NiTe_2_/MoS_2_ [31], and TiO_2_/MoS_2_ [32] were successfully synthesized. In-plane 1T-/2H-MoS_2_ heterostructures were experimentally and theoretically studied, and were found to be effective for ion storage, photodegradation, and hydrogen evolution reaction [33,34,35]. Furthermore, the electronic properties of MoS_2_/BN and MoS_2_/G, MoS_2_/MoSi_2_N_4_ layered heterostructures were investigated under an external electric field and a strain potential [36].

Hybrid structures can be built between 1D and 2D material platforms (no constraint of lattice matching) such as carbon nanotubes with MoS_2_ monolayer [37]. A hybrid of Au nanoflakes mixed with MoS_2_ monolayer was prepared [38]. Additionally, the ZnO nano particles on the MoS_2_ monolayer were synthesized and the enhanced Raman and photoluminescence emissions were observed [39]. Additionally, the composite of MXene-Graphene/Hexagonal-Boron Nitride Structures was recently studied [40].

In this work, we investigate the structure and electronic properties of hybrid G/BN@2H-MoS_2_ monolayers using first principles calculations. The hybrid structures are constructed from a monolayer of 2H-MoS_2_ with an embedded patch of G or BN in the Mo or S layers. We also investigate the adsorption of H_2_, OH, N_2_, NO, CO, CO_2_, NO_2_, H_2_S, SO_2_, COOH, CH_4_, and NH_3_ on the considered hybrid structures.

## 2. Computational Methods

All calculations are performed using density functional theory (DFT) on the basis of the projector augmented wave method (quantum espresso package) [41]. First, the energies and wave functions are calculated within the generalized gradient approximation (the Perdew–Burke–Ernzerhof exchange–correlation functional) [42]. The cell-volume and ionic position relaxations of all structures are carried out until all the atomic forces on each ion are less than 10^−4^ eV/Å. A vacuum region of ~ 16 Å is used to avoid the interaction between the layers in the z-direction. We use norm-conserving pseudopotentials with a 50 Ry energy cutoff and a 9 × 9 × 1 k-point grid. The valence electron configurations 4s^2^ 4p^6^ 4d^5^ 5s^1^ for Mo and 3s^2^ 3p^4^ for S atoms are used to calculate their potentials. The Van der Waals correction is considered [43]. A 6 × 6 × 1 G/BN@MoS_2_ supercell is created by embedding a patch of graphene/BN into MoS_2_ monolayer. Löwdin charges are used to calculate the charge transfer between the monolayers and molecules. Spin-polarized calculations show that all the considered heterostructures in this study are nonmagnetic. The stability of the considered hybrid structures is estimated by calculating their formation energies (*E^form^*) using the following equation:

(1) $$E^{form}=\frac{E^{hyb}-E^{MoS}-E^{pat}}{n^{hyb}}$$

where *E^hyb^* and *E^MoS^ (n^Mo^E^Mo^ + n^S^E^S^)* are the total energy of the hybrid sheet and the total energy of the constituent atoms of the sheet, respectively, with a total number of the atoms in the hybrid structure (*n^hyb^)*. The energy of the patch is *E^pat^ = n^C^ E^C^* for the G-patch and *E^pat^ = n^B^ E^B^ + n^N^E^N^* for the h-BN-patch, where *n^x^* is the total number of the *x* atom (*x = Mo*, *S*, *C*, *B*, *N*). The adsorption energy (*E^ads^*) of a molecule on a sheet is calculated by:

(2) $$E^{ads}=E^{sheet+molecule}-E^{sheet}-E^{molecule}\;,$$

where *E^sheet+molecule^*, *E^sheeat^* and *E^molecule^* are the total energies of the sheet with the adsorbed molecule, the sheet, and the isolated molecule, respectively.

## 3. Structural and Electronic Properties of Hybrid Monolayers

To investigate the effect of G and BN patches on the electronic properties of the MoS_2_ sheet, we first calculate the structural parameters and electronic properties of the pristine MoS_2_ sheet to establish a reference. The lattice constant of the optimized structure is 3.22 Å with an S-Mo bond length of 2.42 Å, which agrees well with the corresponding experimental values of 3.18 Å and 2.41 Å (2.41 Å) [44].

**Figure 1:**
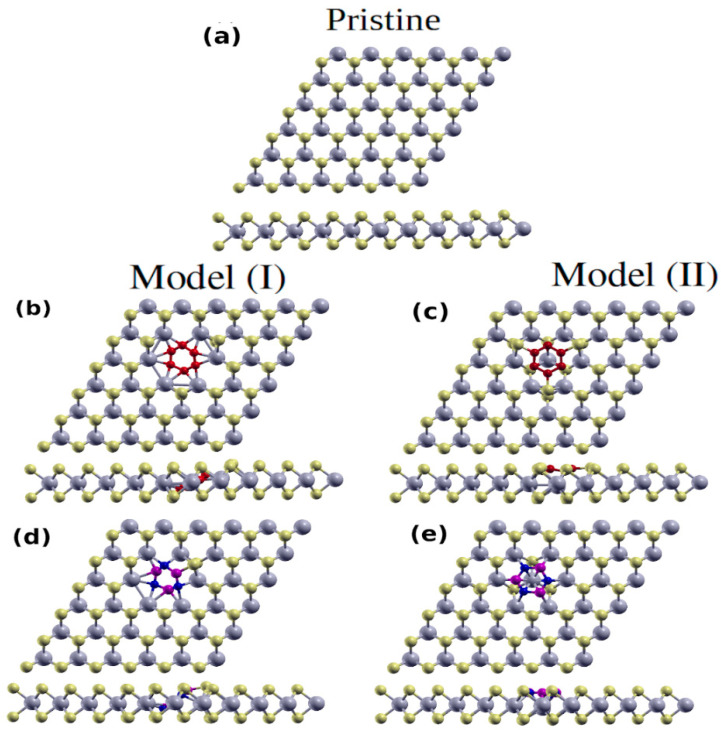
Top and side views of (**a**) pristine optimized structures of MoS_2_, (**b**) G@MoS_2_ model I, (**c**) MoS_2_ model II, (**d**) h-BN@MoS_2_ model I, and (**e**) h-BN@MoS_2_ model II.

Now, we investigate the effect of G-patch on the structural properties of the MoS_2_ sheet. Interface models with different edge terminations (Mo or S) have been considered [45]. The electronic properties are found to be strongly dependent on the termination, which can be correlated with the existence of polar C-Mo bonds or defects caused by the C-S bonds at the interface. The two relaxed G@2H-MoS_2_ structures are shown in (Figure 1b,c). The first hybrid structure (Figure 1b), built by removing one Mo atom and six S atoms has the G-patch connected to Mo atoms. The other hybrid structure is created when the G-patch is formed in the S layer, after the removal of 3 S atoms, giving a total number of 111 atoms in the supercell (Figure 1c). The patch’s boundary constitutes the main defect in the MoS_2_ monolayer, which may change the physical and/or chemical properties of MoS_2_. Structural relaxation yields a local symmetric distortion in the Mo sites surrounding the carbon atoms.

The Mo–Mo distance decreases from 3.22 Å to 3.02 Å with no significant change in the Mo-S bonds (~2.41 Å) for hybrid and pristine cases. The C-C bond length in the hybrid structures is 1.42 Å (1.47 Å) for the first (second) model, which is close to the corresponding value in pristine graphene (1.42 Å). The average C-Mo and C-S lengths are 2.13 Å and 1.79 Å, respectively, which are smaller than the S-Mo of 2.41 Å due to the small size of the C atom compared to the S atom.

Turning to the first h-BN@MoS_2_ hybrid structure (Figure 1d), we see that it is also slightly buckled with ~0.35 Å, especially inside the BN patch. The average bond length for Mo–Mo decreases from 3.21 Å to 3.05 Å. The bond length of B-N in the structure is 1.45 Å, which is the pristine h-BN value [5]. We find the optimized bond lengths for N-Mo and B-Mo bonds are 2.14 Å and 2.09 Å, respectively. These are smaller than S-Mo (2.41 Å) due to the small size of the B and N atoms compared to the S atom. The second hybrid structure (Figure 1e) has an optimized B-N bond length of 1.47 Å, while the N-Mo and B-Mo bond lengths are 2.21 Å and 2.27 Å, respectively—larger than N-Mo and B-Mo bonds of the first configuration. We notice that no buckling occurs, which causes the Mo–Mo bond length to be 3.21 Å. We calculate the formation energy *E^form^* of the hybrid structures using Equation (1) and find the pristine energy to be −7.09 eV, while the energies of the hybrid structures range between −7.14 and −7.17 eV, reflecting the stability of the studied hybrid structures.

**Figure 2:**
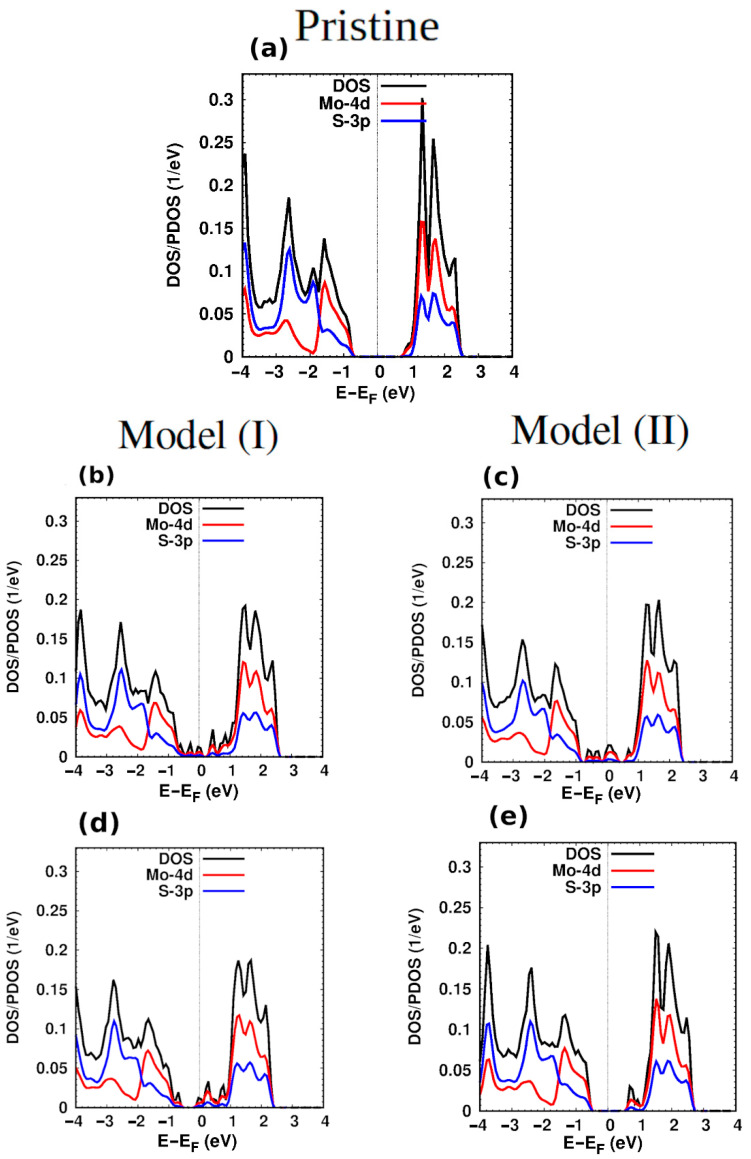
DOS/PDOS of (**a**) pristine MoS_2_, (**b**) G@MoS_2_ model I, (**c**) G@MoS_2_ model II, (**d**) h-BN@MoS_2_ model I, and (**e**) h-BN@MoS_2_ model II.

Now, we discuss the electronic properties of the hybrid structures. To establish a reference, we first discuss the properties of the pristine MoS_2_. The DOS of pristine MoS_2_ is shown in (Figure 2a). The band gap is 1.73 eV, which is in good agreement with the reported experimental (theoretical) value of 1.80 eV (1.74 eV) [44,46,47]. The electronic states near the top of the valence band and the bottom of the conduction band are mainly composed of Mo states which agree with the previous literature [48]. Figure 2b,c show the DOS/PDOS of hybrid structures (models I and II) of G@2H-MoS_2_. The G-patch makes the semiconducting MoS_2_ metallic sheet. The hexagonal carbon patch disturbs the DOS of the MoS_2_ for both hybrid structures, giving rise to midgap states that cover most of the bandgap region of MoS_2_. The DOS is also shifted towards lower energy compared to the pristine structure. The Mo states are dominant among the midgap states (Figure 2b). The effect of the G-patch in the second configuration, (Figure 2c), is very similar to the first configuration. At the Fermi energy, the density of states of the second model is larger than the corresponding states of the first model, which can be attributed to the number of Mo and S atoms in the second model being larger than in the first model. The DOS of the first hybrid structure of h-BN@MoS_2_ is shown in Figure 2d. The Fermi energy is shifted towards the bottom of the conduction band, which means the hybrid structure is n-type conducting. The created state narrows the band gap to 0.72 eV compared to the bandgap of the pristine structure. The Mo states are dominant in the energy range from −1.8 to 2.4 eV. The top of the valence band and bottom of the conduction band are disturbed compared to the pristine structure due to the h-BN-patch. For the second configuration (Figure 2e), the effect of the BN-patch is very similar to the pristine (Figure 2a), which means the hybrid structure is semiconducting, similar to the pristine. The disturbance appears only at the edge of the conduction band due to the created states. The Mo states prevail in the energy range of −1.8 to 3.3 eV. The bandgap becomes 1.2 eV, which is smaller than that of the pristine and larger than that the corresponding value for the first hybrid configuration.

## 4. Molecular Adsorptions

**Figure 3:**
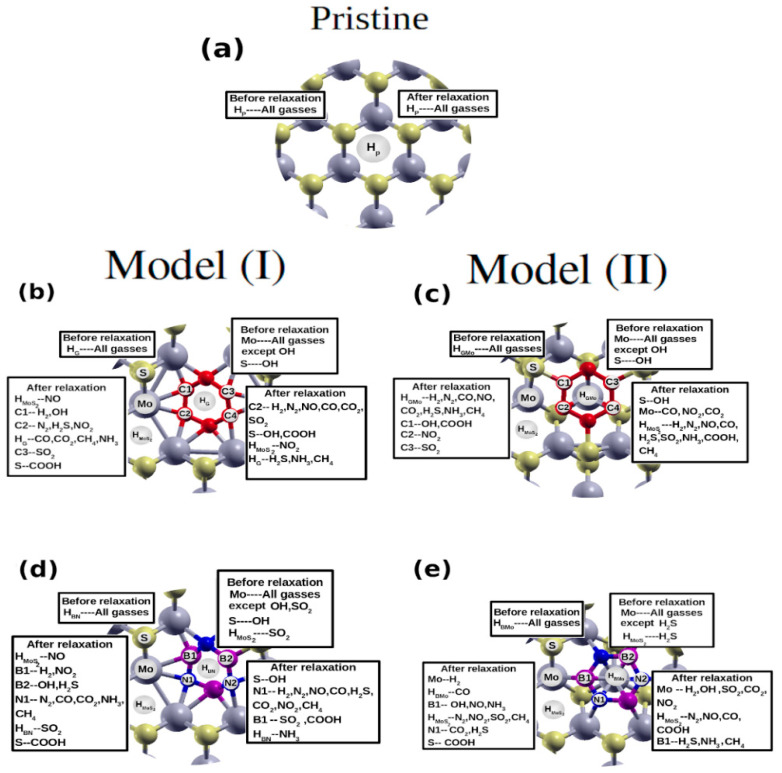
The positions of the adsorbed molecules before and after optimization of (**a**) pristine MoS_2_, (**b**) G@MoS_2_ model I, (**c**) G@MoS_2_ model II, (**d**) h-BN@MoS_2_ model I, and (**e**) h-BN@MoS_2_ model II.

Now, we discuss the adsorption properties of the hybrid sheets compared to the pristine MoS_2_. We consider the following gases: diatomic (OH, NO, CO, N_2_, H_2_), triatomic (NO_2_, H_2_S, SO_2_, CO_2_), tetratomic (COOH, NH_3_), and polyatomic (CH_4_). The relaxed structures are used with distinct starting sites for the adsorption. For the pristine MoS_2_, we place the gas on the top of the hollow site (H_P_), (Figure 3a), which has been shown to be the most favorable site [49]. For the hybrid structures, we used two starting positions: the first position on top of the hexagon center of the G/h-BN-patch (H_G_/H_BN_) for the first configuration (Figure 3b,d) and (H_GMo_/H_BMo_) for the second configuration (Figure 3c,e). The starting and ending locations for all adsorbed molecules are shown in Figure 3a–e.

We started the relaxation from multiple initial positions close to the hexagon center of the G/BN patch, as well as on the pore edge (including the Mo and S atoms at the edge, Figure 3). We find that for some molecules, there are multiple final positions. The adsorption energies of various final locations are discussed below. The adsorption energy *E^ads^* is calculated using Equation (2).

**Figure 4:**
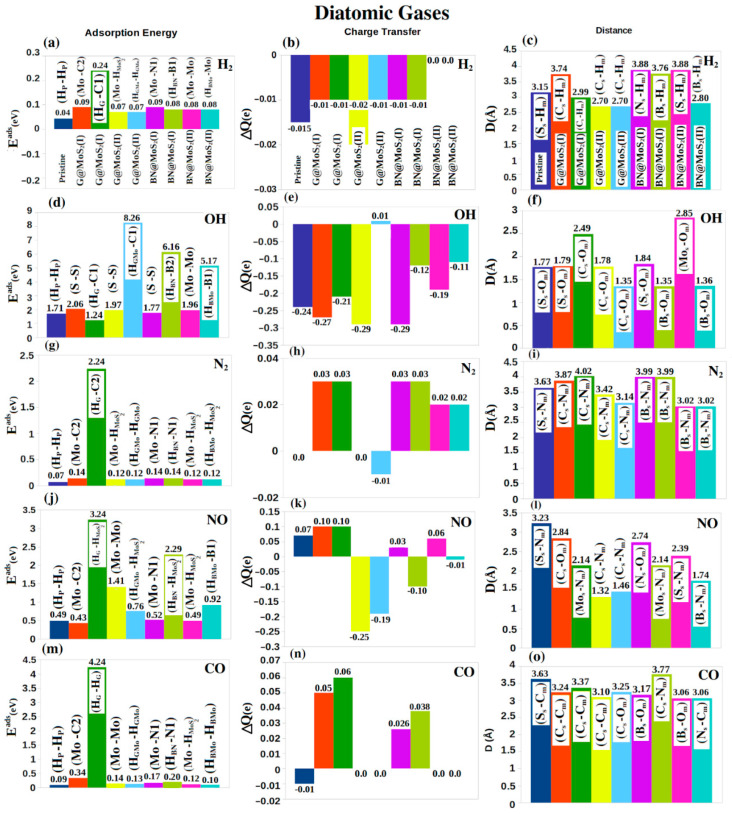
(**a**–**o**) From left to right, the adsorption energy (*E^ad^* (eV)), charge transfer (ΔQ (e)) and the and closest distance (D(Å)) between the gas (X_m_) and the sheet (X_s_) for diatomic gases. From top to bottom H_2_, OH, N_2_, NO, and CO gases are considered. The initial and final positions (Y, Z) of the adsorbed gases before and after optimization are shown in the adsorption energy figures (first column).

In Figure 4, we show the adsorption results of four molecules. From left to right, the subfigures show the adsorption energy (first column), the charge transfer between the sheet and the adsorbent (second column), and the shortest distance between the adsorbent atom and the sheet atom (third column). Each subfigure considers nine cases: the pristine, and four hybrid systems, each with two starting locations for the adsorbent. The starting and ending locations are shown on the adsorption energy subfigures above the bar representing the adsorbent, while the shortest distance between the adsorbent atom and the sheet atom is shown in the corresponding bar in the distance subfigure.

We first report the bond lengths of the isolated adsorbents. These are 0.75 Å and 0.98 Å for H_2_ and OH, respectively, which are similar to experimental values of 0.74 Å [50] and 0.97 Å [51], respectively. After structural optimization, the bond lengths of both molecules do not change for the pristine and the hybrid structures. The bond lengths of the isolated N_2_, NO, and CO are 1.09 Å, 1.16 Å, and 1.14 Å, respectively, similar to experimental bond lengths (1.10 Å, 1.15 Å, and 1.13 Å [51,52], respectively). The bond lengths of these molecules do not change on the hybrid structures except for the NO molecule on G@MoS_2_ (II), where the N-O length increases to 1.27 Å.

For H_2,_ we find the largest adsorption energy, 0.24 eV at C1, is on the G@MoS_2_ (II) sheet. All other hybrid sheets adsorb the H_2_ molecule by approximately 0.08 eV. Our results are very close to the previous published results for pristine MoS_2_ (0.06 eV) [53] and pristine G (0.08 eV) [54].

For the OH molecule, it is physisorbed on the pristine MoS_2_ sheet with 1.71 eV, in agreement with published results [55]. On the other hand, it is chemisorbed on G@MoS_2_ (II) at (H_GMo_, C1), B@MoS_2_ (I) at (H_BMo_, B2), B@MoS_2_ (II) at (H_BMo_, B1), and G@MoS2 (I) at (S, S) (Figure 4d), with energies of 8.26 eV, 6.16 eV, 5.17 eV and 2.06 eV, respectively. The closest distances from the sheets are 1.35 Å between C_s_ and O_m_, 2.85 Å (Mo_s_-O_m_), 1.36 Å between B_s_ and O_m_, and 1.79 Å (S_s_-O_m_), respectively (Figure 4f), where the subscripts “s” and “m” refer to the sheet and molecule, respectively.

The charge transfer from the sheet to the OH is largest (~−0.29*e*) for G@MoS_2_ (I)/(II) and BN@MoS_2_ (I), which adsorb OH at S atom as the initial and final position with the closest distance of 1.35 Å (Figure 4e), while it is −0.19*e* for BN@MoS_2_(II). Note that the length of O-H bond remains unchanged for all considered systems. The charge transfer from the sheets to the OH matches the nature of OH as an acceptor group. The hybrid G@MoS_2_ (I) can thus be used for oxygen reduction reaction under certain conditions [56].

Our calculated N-N bond length (1.10 Å) is close to the previous reports (1.09 Å) [57], and it does not change significantly when we add it to our considered sheets. The G@MoS_2_ (I) structure chemisorbs N_2_ at (H_G_, C2) (Figure 4g) with 2.24 eV and a distance of 4.02 Å (C_s_-N_m_) (Figure 4i). All other hybrid structures physisorb N_2_ weakly with an average adsorption energy of ~0.13 eV, which is slightly larger than the adsorption energy on the pristine system (0.07 eV) (Figure 4g). The charge transfer from the molecule to most hybrid structures is ~0.03*e* (Figure 4h).

The last two diatomic gases are NO and CO. The bond lengths of the isolated NO, CO are 1.16 Å, and 1.14 Å, respectively, which match published values (1.15 Å and 1.13 Å [58,59]). The N-O bond slightly increases to 1.20 Å for most of the considered structures while the C-O bond shows no significant change. The largest adsorption energy is 3.24/4.24 eV for NO/CO on the G@MoS_2_ (II) at (H_G_, H_MoS2_)/(H_G_, H_G_), (Figure 4j,m), with charge transfer of 0.1/0.06*e*, (Figure 4k,n), and at a distance of 2.14/3.37 Å for (Mo_s_-N_m_)/(C_s_-C_m_) bonds, (Figure 4l,m). The NO is also chemisorbed on BN@MoS_2_ (I) at (H_BN_, H _MoS2_) with energy of 2.29 eV, (Figure 4j), a charge transfer of −0.1*e*, (Figure 4k), and at a distance of 2.14 Å (Mo_s_-N_m_) (Figure 4l). The pristine and hybrid structures thus adsorb NO more strongly than CO. The adsorption of NO/CO on the hybrid structures is generally stronger than that of graphene (0.03/0.01 eV [60]) and h-BN (0.03/0.02) eV [61]. Adsorption of NO/CO on the hybrid systems is also superior to that on MoS_2_ doped Au, Pd, Pt, and Ni (1.62/1.38 eV for NO/CO [62]), which indicates that our hybrid systems may be considered for NO/CO filtration. Summarizing our results for the diatomic molecules, the hybrid structures (especially G@MoS_2_ (I)) are suitable for the physisorption and chemisorption for N_2_, NO, and CO. However, all the considered structures adsorb the H_2_ very weakly. For charge transfer, OH and H_2_ act as acceptors, while N_2_ and CO act as donors.

**Figure 5:**
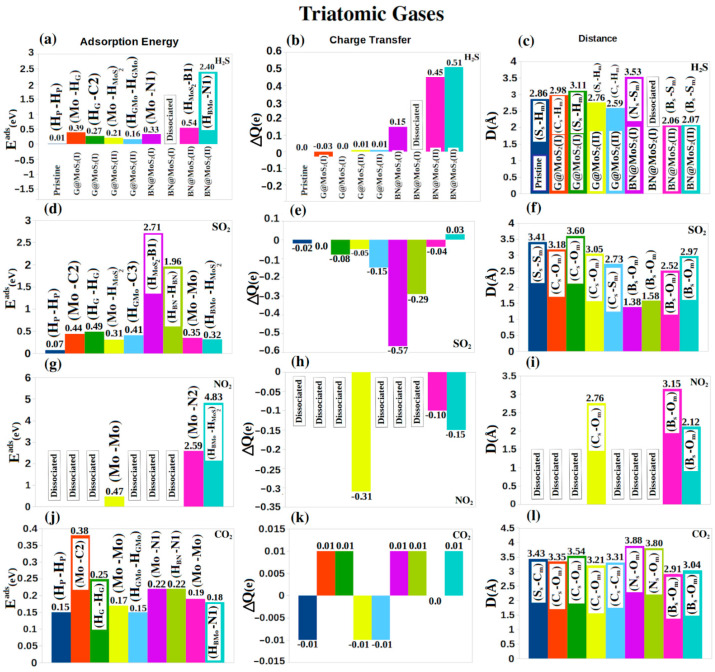
(**a**–**l**) From left to right, the adsorption energy (*E^ad^* (eV)), charge transfer (ΔQ (e)) and the closest distance (D(Å)) between the gas (X_m_) and the sheet (X_s_) for triatomic gases. From top to bottom H_2_S, SO_2_, NO_2_, and CO_2_ gases are considered. The initial and final positions (Y, Z) of the adsorbed gases before and after optimization are shown in the adsorption energy figures (first column).

We now move to triatomic gases and begin our discussion by H_2_S. Our calculated H–S bond length and H-S-H angle for the isolated molecule are 1.37 Å and 92.1°, in good agreement with the corresponding published values (1.34 Å and 92.1° [63]). For adsorption on most systems, we observe no significant change in the bond length, while the angle slightly increases to approximately 92.5°. BN@MoS_2_ (II) at (H_BN_, N1) adsorbs the H_2_S chemically with the energy of 2.40 eV (Figure 5a) with a large charge transfer of 0.51*e*, (Figure 5b), and the distance of 2.07 Å (B_s_-S_m_) (Figure 5c). H_2_S dissociates on BN@MoS_2_ (II) while it is physisorbed on the remaining gases.

For SO_2_, the bond length S–O and the O–S–O angle are 1.48 Å and 120.2°, respectively, in good agreement with published values (1.43 Å and 119.5° [64]). When we place SO_2_ on the considered sheets the bond length does not change but the angle changes to (118.97°, 116.82°) for G@MoS_2_ (I), (118.33°, 117.67°) for G@MoS_2_ (II), (100.46°, 112.15°) for BN@MoS_2_ (I) and (118.00°, 118.59°) for G@MoS_2_ (II) at the two possible final positions of the molecule. SO_2_ is chemisorbed by BN@MoS_2_(I) at (H_MoS2_, B1) with an energy of 2.71 eV (Figure 5d), a charge transfer of –0.57*e* (Figure 5e), and a distance of 1.38 Å (B_s_-O_m_) (Figure 5f). The largest physisorption for H_2_S is on BN@MoS_2_ (I) at (H_BN_, H_BN_) with energy, charge transfer, and distance of 1.96 eV, –0.29*e* and 1.58 Å (B_s_-O_m_), respectively, (Figure 5d–f). 

For NO_2_, the bond length and the angle of the isolated molecule are 1.21 Å and 133.5°, respectively, which agree with the reported values (1.20 Å and 134.3° [65]). On G@MoS_2_ (II), NO_2_ has a bond length of 1.24 Å and an angle of 125.1°. It is physisorbed at (Mo, Mo) with an energy of 0.47 eV, a charge transfer of −0.31*e*, and a distance of 2.76 Å (C_s_-O_m_) (Figure 5h,i). On BN@MoS_2_ (II), the bond length and angle are reduced to (1.22 Å,130.00°) and (0.93 Å,106.30°) for its two instances, (Mo, N_2_) and (H_BMo_, H_MoS2_). The molecule is chemisorbed in both cases with energies of 2.59 and 4.83 eV (Figure 5g), charge transfers of –0.1*e* and –0.15*e (*Figure 5h) and (B_s_-O_m_) distances of 3.15 and 2.12 Å (Figure 5i), respectively. On all other systems, NO_2_ dissociates.

The last triatomic gas we study is CO_2_. Our calculated C-O bond length and O-C-O angle for the free gas are 1.18 Å and 180° (the corresponding literature values are 1.16 Å and 180°.00 [65]). There is no significant change in the bond length and the angle when we add the CO_2_ to the different studied systems. The molecule is weakly physisorbed on all structures, with an energy of a fractional eV. The adsorption energy ranges from 0.15 to 0.38 eV, which is better than the corresponding values (0.14 to 0.25 eV) obtained for 2H-@MoS2 nanosheets, nanotubes, and nanopores [66].

**Figure 6:**
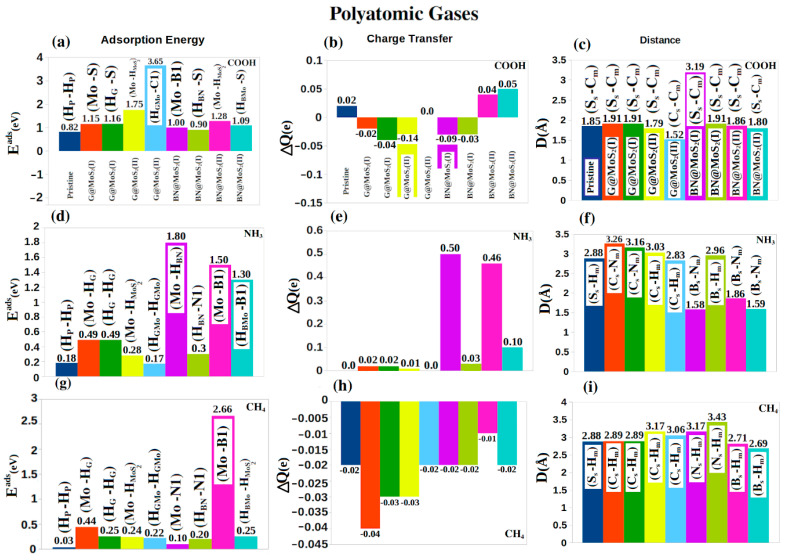
(**a**–**i**) From left to right, the adsorption energy (*E^ad^* (eV), charge transfer (ΔQ (e) and the closest distance (D(Å)) between the gas molecule (X_m_) and the sheet (X_s_) for polyatomic gases. From top to down COOH, NH_3_, and CH_4_ gases are considered. The initial and final positions (Y, Z) of the adsorbed gases before and after optimization are shown in the adsorption energy figures (first column).

Turning to the last gases group, polyatomic gases (COOH, NH_3_, and CH_4_), the carboxyl molecule COOH (Figure 6a–c) has an angle (∠O-C-O) of 131.23° with two bond lengths of 1.19 Å and 1.34 Å [67]. When the COOH is adsorbed on the pristine and hybrid sheets, the angle slightly changes to: 129.47° for pristine, (128.58°, 127.88°) for G@MoS_2_(I), (125.50°, 125.50°) for G@MoS_2_(II), (124.96°, 124.90°) for BN@MoS_2_(I), and (129.90°, 129.71°) for BN@MoS_2_ (I), at the two different final positions for every hybrid structure. However, the bond lengths of COOH did not change significantly. We also notice that the molecule is closest to the sheet with a Cs-Cm distance of 1.52 Å for the G@MoS_2_(II) system (Figure 6c). In all cases, the charge transfer with the sheets is very weak. Most importantly, COOH is chemically adsorbed by G@MoS_2_ (II) at (H_GMo_, C1) with an energy of 3.65 eV without any charge transfer (indicating covalent bond). All other hybrid sheets physically adsorb the COOH gas with an average energy of ~1.2 eV. Our calculations show that the adsorption energy of COOH reaches 1.75 eV on our hybrid structures compared to 1.6 on hybrid 1T-@2H-MoS_2_ monolayer which may be utilized in many applications such as the decomposition of organic dyes [34]. 

Regarding the isolated NH_3_ and CH_4_ gases, the bond length and angles are 107° and 1.02 Å for NH_3_ [68], and 109.47° and 1.10 Å for CH_4_ [68]. When they are adsorbed on the considered sheets, the bond lengths and the characteristic angles of these molecules do not significantly change compared to the corresponding isolated cases. The largest physisorption for NH_3_ (Figure 6d) is on BN@MoS_2_ (I) with1.80 eV at (Mo, H_BN_) with a distance of 1.58 Å (B_s_-N_m_) and a charge transfer of 0.50*e* (Figure 6e,f). BN@MoS_2_ (I) improves the adsorption energy of NH_3_ compared to pristine G and h-BN (0.03 eV [60,61]). For CH_4_, the most significant adsorption energy is 2.66 eV for BN@2H-MoS_2_(II) at (Mo, B1) and the closest distance between the molecule and the sheet is 2.71 Å (C_s_-H_m_) with a charge transfer of −0.04*e* (Figure 6g–i). All considered monolayers adsorb them with energy less than 2.00 eV (physisorption). To summarize, the adsorption energies of COOH are larger than those of NH_3_ and CH_4_ of the ability to redistribute the charge over the length of COOH. We also notice that NH_3_ acts as a donor, which matches some experimental results of NH_3_ adsorption on a G sheet [69].

Although we have considered hybrid structures with a small G/BN patch, we expect our results to be applicable to structures with bigger patches of various shapes. This is because the adsorption properties largely depend on the structural defects at the border of the G/BN patch rather than the inner part of the patch. The adsorption capacity will vary with the G/BN concentration but will roughly depend on the square root of the concentration.

## 5. Conclusions

In this work, first principle calculations are employed to study the structure, electronic, and molecular adsorption properties of graphene/hexagonal boron nitride@2H-molybdenum disulfide (G/BN@MoS_2_) monolayers. We consider systems where the G/BN patch is at the Mo plane (model I) and the S plane (model II). The G@MoS_2_ systems are metallic, while the BN@MoS_2_ (I) is n-type semiconducting, and BN@MoS_2_ (II) is semiconducting. Compared to the pristine G, BN, and MoS_2_, the hybrid systems have higher adsorption energies for the considered gases (diatomic gases: H_2_, OH, N_2_, NO, CO, triatomic gases: CO_2_, NO_2_, H_2_S, SO_2_, and polyatomic gases: COOH, CH4, and NH_3_). OH is physisorbed on G@MoS_2_ (I,II), which can be used for oxygen reduction reactions. NH_3_ is physisorbed on BN@MoS_2_ (I,II), making them a suitable material for NH_3_-based hydrogen production. H_2_, CO_2_, and CH_4_ are weakly physisorbed on all hybrid structures. We also find that chemisorption occurs for: NO and CO on G@MoS_2_ (I), SO_2_ on BN@MoS_2_ (I), and H_2_S and NO_2_ on BN@MoS_2_ (II), which makes these hybrid systems suitable for use as filter materials for these toxic gases.

## Data Availability

The data presented in this study are available in the article.
